# Shared Immunopathogenic Mechanisms in Chronic Spontaneous Urticaria, Vitiligo, and Hashimoto’s Thyroiditis: The Role of Oxidative Stress and Vitamin D

**DOI:** 10.3390/life15101535

**Published:** 2025-09-29

**Authors:** Rossella Casella, Federica Li Pomi, Francesco Borgia, Eustachio Nettis, Sebastiano Gangemi

**Affiliations:** 1 Department of Emergency and Organ Transplantation, School of Allergology and Clinical Immunology, University of Bari Aldo Moro, 70126 Bari, Italy; rossellacasella7@gmail.com (R.C.); ambulatorio.allergologia@uniba.it (E.N.); 2 Department of Precision Medicine in Medical, Surgical and Critical Care (Me.Pre.C.C.), University of Palermo, 90127 Palermo, Italy; 3 Section of Dermatology, Department of Clinical and Experimental Medicine, University of Messina, 98125 Messina, Italy; fborgia@unime.it; 4 Department of Clinical and Experimental Medicine, School and Operative Unit of Allergy and Clinical Immunology, University of Messina, 98125 Messina, Italy; sebastiano.gangemi@unime.it

**Keywords:** oxidative stress, vitamin D, urticaria, vitiligo, Hashimoto thyroiditis, phototherapy, redox balance, malondialdehyde

## Abstract

Introduction: Chronic spontaneous urticaria (CSU), vitiligo, and Hashimoto’s thyroiditis (HT) frequently co-occur in the same patients, suggesting a shared autoimmune pathogenesis. These conditions are increasingly recognized as components of polyautoimmunity, with overlapping clinical, immunological, and pathogenetic features. Among the proposed common mechanisms, vitamin D deficiency and oxidative stress (OS) have emerged as key contributors. We aimed to explore the shared immunopathogenic pathways linking these conditions, with a focus on the interplay between vitamin D status and redox imbalance. Methods: An extensive narrative review of the current literature regarding the associations among CSU, vitiligo, and HT, focusing on the role of vitamin D status, OS, and nitrosative stress, and shared immunological pathways was conducted. Discussion: Vitamin D deficiency was consistently observed across all three conditions and is associated with increased disease activity and poorer clinical outcomes. Several polymorphisms in the vitamin D receptor (VDR) and binding protein genes correlate with disease susceptibility. OS and nitrosative stress markers, such as malondialdehyde (MDA) and nitric oxide (NO) metabolites, are elevated in patients with CSU, vitiligo, and HT, and are linked to tissue-specific immune activation, apoptosis, and loss of self-tolerance. Evidence suggests that vitamin D and antioxidant supplementation may provide clinical benefit. In vitiligo, narrowband ultraviolet B (NB-UVB) phototherapy not only promotes repigmentation through melanocyte stimulation but also reduces ROS production and modulates local immune responses. Conclusions: The coexistence of CSU, vitiligo, and HT reflects a broader systemic autoimmune tendency, with vitamin D deficiency and redox imbalance serving as potential unifying mechanisms. Routine assessment of vitamin D levels and OS parameters may enhance diagnostic precision and inform therapeutic strategies. Antioxidant-based interventions represent promising avenues in the integrated management of autoimmune skin and endocrine disorders.

## 1. Introduction

Polyautoimmunity, namely the coexistence of two or more autoimmune diseases in a single patient, suggests the existence of intricate underlying mechanisms driving common pathogenesis, including the interplay between genetic susceptibility, environmental factors, and immune dysregulation. 

In this scenario, chronic spontaneous urticaria (CSU), vitiligo, and Hashimoto’s thyroiditis (HT) often co-occur, reflecting a common pathogenetic background with clinical and immunological implications that extend beyond the single presentations of each disease. Vitiligo, characterized by depigmented patches with sharp margins, is caused by the destruction of melanocytes through autoimmune mechanisms, genetic factors, and oxidative stress (OS), affecting up to 1–2% of the population [1]. Approximately 34% of vitiligo patients present high levels of thyroid autoantibodies, including anti-thyroglobulin (ATG) and anti-thyroid peroxidase (TPO), even without evident thyroid dysfunction, which suggests a potential pathogenetic role of these antibodies [2,3,4]. In addition, studies have shown that patients with vitiligo have a higher prevalence of chronic autoimmune thyroiditis, with an increased risk of 1.7 to 2.5 times compared to the general population [5].

HT, also known as chronic lymphocytic or autoimmune thyroiditis, is the most common organ-specific autoimmune disease, which is caused by increased thyroid lymphocytic infiltration and the presence of antibodies specific to thyroid antigens, resulting in progressive dysfunction, ranging from subclinical hypothyroidism to overt hypothyroidism. It is clinically characterized by local and systemic symptoms, including fatigue, weight gain, cold intolerance, and hair disorders [6]. This disease is frequently associated with a plethora of autoimmune disorders, including the already mentioned vitiligo and CSU. 

CSU has been linked to autoimmune thyroid disorders, with approximately 25% to 30% of CSU patients testing positive for TPO antibodies, highlighting a significant association between these conditions. Specifically, CSU, presenting as recurrent flares of wheals and/or angioedema persisting for over six weeks, is characterized by high serum levels of autoantibodies against the high-affinity immunoglobulin E (IgE) receptor or IgE itself, which suggests a similar underlying autoimmune etiology. Looking at it from another perspective, HT is present in more than 5% of CSU patients, while vitiligo is present in more than 3% of cases [7].

These three conditions can also cluster in patients, exemplifying the concept of “polyglandular autoimmune syndrome type IIIC,” which encompasses both endocrine and non-endocrine disorders. Clinically, these patients may present with CSU, depigmented patches characteristic of vitiligo, and symptoms of thyroid dysfunction with antibody positivity [8].

Shared immunological pathways, including CD8+ T cell dysregulation, reactive oxygen species (ROS) imbalance, and common heritable susceptibility genes, seem to drive the pathogenesis of these apparently so different but interlinked diseases. ROS-induced OS seems to be a mutual denominator, damaging melanocytes and thyroidocytes and inducing mast cell degranulation, thereby perpetuating immune activation, autoantibody production, and tissue destruction [9,10,11].

In light of these considerations, screening for thyroid autoantibodies in patients with vitiligo or CSU has been recommended, as early identification of thyroid dysfunction can improve patients’ management [12]. Similarly, dermatological evaluation for vitiligo and CSU in patients affected by thyroiditis can guide a more holistic approach to patient care. The integration of clinical recognition with biochemical and immunological assessments is essential to early identify these comorbidities, thus ensuring patient-tailored treatments and effective management strategies.

Moving from these premises, we aimed to provide an updated overview of the common immunopathogenic pathways linking CSU, vitiligo, and HT, with a specific focus on the role of vitamin D deficiency and OS in disease pathogenesis.

## 2. Materials and Methods

A narrative literature review was conducted through a comprehensive search of the PubMed database, without date restrictions, up to 10 June 2025. We used the following keyword combinations: “vitiligo AND oxidative stress,” “vitiligo AND vitamin D,” “chronic spontaneous urticaria AND oxidative stress,” “chronic spontaneous urticaria AND vitamin D,” “Hashimoto’s thyroiditis AND oxidative stress,” and “Hashimoto’s thyroiditis AND vitamin D”. Additional studies were identified by screening the reference lists of the selected papers. We included original clinical and experimental studies, reviews, and meta-analyses addressing the associations among vitamin D status, OS, and the pathogenesis of CSU, vitiligo, and HT. Studies not directly relevant to these outcomes or not available in English were excluded.

## 3. Discussion

### 3.1. Low Level of Vitamin D and Its Role in the Pathogenesis of Chronic Urticaria, Vitiligo, and Autoimmune Thyroiditis

Vitamin D, a fat-soluble vitamin, exists in two forms, D2 (ergocalciferol) and D3 (cholecalciferol), and is acquired mainly from skin activation after sun exposure and only a small amount from diet [13,14]. Vitamin D deficiency, generally defined as 25-hydroxyvitamin D [25(OH)D] levels less than 20 ng/mL, is a major public health problem [15], affecting 30–60% of children and adults worldwide [16]. Potential factors influencing vitamin D status include oral vitamin D intake, sun exposure, latitude, season, Fitzpatrick skin type, sun exposure practices, body mass index (BMI), physical activity, alcohol consumption, and genetic polymorphisms. Besides its major role in mineral homeostasis and bone physiology [15], vitamin D plays a crucial role in the immune system, activating both innate and adaptive immune responses [17] by binding to its nuclear receptors, the vitamin D receptor (VDR), and plasma membrane receptors on epithelial cells, as well as various other cell types, including mast cells, monocytes, macrophages, T-cells, B-cells, and dendritic cells. It enhances defense mechanisms against microbes by stimulating the expression of antimicrobial peptides such as cathelicidin and defensins [16]. Vitamin D is also involved in decreasing excessive inflammation by suppressing Toll-like receptor (TLR) production by monocytes, enhancing mast cell production of interleukin (IL)-10, an anti-inflammatory cytokine, inhibiting dendritic cell activation by lipopolysaccharide (LPS), decreasing cytokine secretion from T helper (Th) 1 cells, inducing regulatory T cells, and inhibiting B lymphocyte function and IgE secretion [13]. Notably, such immunomodulatory effects are compromised not only in conditions of overt deficiency but also when serum concentrations remain within the so-called suboptimal range (20–30 ng/mL). In these “low-level” states, vitamin D may be less effective in sustaining regulatory T cell activity and controlling Th1/Th17 polarization, resulting in an increased production of proinflammatory cytokines and impaired immune tolerance [14]. This intermediate insufficiency may therefore act as a permissive factor for chronic immune activation, contributing to the pathogenesis of autoimmune and inflammatory diseases, including CSU, vitiligo, and HT [18,19].

#### 3.1.1. Correlation Between CSU and Vitamin D Serum Levels

Low serum concentrations of 25(OH)D have been associated with several dermatological conditions, including severe atopic dermatitis [20], psoriasis [21], and acne [22], and CSU. In CSU, it has been postulated that insufficient vitamin D levels may impair immune cell function, resulting in elevated circulating proinflammatory cytokines and dysregulation of T regulatory cell cytokine profiles, thereby contributing to the exacerbation of the symptoms. Vitamin D exerts its biological effects through interaction with the vitamin D binding protein (VDBP), the primary transport protein in the circulation, and VDR, which mediates genomic responses. The GC gene encodes VDBP, and its genetic polymorphisms influence both the concentration of the protein and its binding affinity for vitamin D [23]. The VDR gene, located on chromosome 12, undergoes epigenetic modulation upon ligand binding, leading to the transcriptional regulation of various vitamin D-responsive genes [24]. Polymorphisms in the VDR, particularly single-nucleotide polymorphisms (SNPs) such as rs1544410 and rs2228570, have been frequently studied in the context of allergic diseases due to their potential impact on receptor function and downstream vitamin D activity [25]. Notably, a positive association between serum VDBP levels and the progression of CSU has been reported, thus suggesting that alterations in the vitamin D pathway at both genetic and protein levels may represent risk factors for CSU development [26]. Several studies have consistently demonstrated that serum 25(OH)D levels are significantly lower in CSU patients compared to healthy controls [13]. Moreover, an inverse relationship has been observed between disease severity and serum 25(OH)D levels, with significantly lower concentrations reported in CSU patients [27,28]. Findings also seem to suggest that vitamin D deficiency may contribute to the progression from acute to chronic urticaria. In support of this, in patients with low vitamin D levels, supplementation was associated with clinical improvement in skin symptoms, including idiopathic pruritus, rash, and urticaria [29,30]. Two meta-analyses published in 2018, conducted by Tsai et al. and Wang et al., further confirmed a significantly higher prevalence of vitamin D deficiency among CSU patients compared to controls [31,32].

#### 3.1.2. Correlation Between Vitiligo and Vitamin D Serum Levels

The role of vitamin D and its receptor polymorphisms in the pathogenesis of vitiligo has gained growing interest in recent years, with accumulating evidence suggesting that variations in the VDR gene may influence serum vitamin D levels and contribute to a genetic predisposition to vitiligo [33]. The interplay between VDR polymorphisms, serum 25(OH)D levels, and vitiligo has become a subject of clinical and epidemiological investigation.

In 2014, a significant association between VDR gene polymorphisms and vitiligo susceptibility was reported, with the ApaI a allele and BsmI bb genotype linked to increased risk in East Asian populations [34]. A subsequent meta-analysis confirmed a correlation between low serum 25(OH)D levels and the onset of vitiligo [35]. Confirming this, oral vitamin D supplementation has been reported to be beneficial in pediatric patients with vitiligo and concomitant vitamin D deficiency [36].

However, results across studies remain inconsistent. Other research found no significant differences in 25(OH)D levels across various VDR genotypes [37], while others reported significantly lower vitamin D levels in vitiligo patients compared to healthy controls, though without correlation to the extent of depigmented patches [38]. Conversely, other studies highlighted no difference in vitamin D levels between patients and controls, and no association with age or disease severity [39].

Vitamin D is known to play a significant role in the biology of both melanocytes and keratinocytes. In vitro studies have shown that vitamin D3 can enhance tyrosinase activity and stimulate melanogenesis, potentially contributing to repigmentation in vitiligo lesions [40]. Topical vitamin D analogs, such as calcipotriol and tacalcitol, have also been reported to promote repigmentation in patients with vitiligo [41]. Beyond its effects on pigmentation, vitamin D exerts pivotal immunomodulatory actions, with the suppression of the expression of key proinflammatory cytokines, including interleukin (IL)-6, IL-8, tumor necrosis factor (TNF)-α, and interferon (IFN)-γ.

Furthermore, the active form of vitamin D appears to reduce ultraviolet B (UVB)-induced apoptosis in melanocytes, offering a potential protective mechanism in vitiligo [42]. Among the various hypotheses proposed for the pathogenesis of vitiligo, the autoimmune theory has gained substantial support. In this context, inflammatory cytokines play a pivotal role in intercellular signaling and immune responses. IL-17, produced by CD4^+^ Th17 cells, is particularly noteworthy for its proinflammatory effects, including the promotion of OS and the impairment of melanocyte function. Vitamin D3 compounds are known not only for their capacity to modulate immune responses but also for their ability to enhance melanogenesis. Several cytokines implicated in vitiligo pathogenesis—including IL-17, IL-2, IL-6, IL-8, TNF-α, and IFN-γ—are regulated, at least in part, by vitamin D and its analogs [43].

#### 3.1.3. Correlation Between Autoimmune Thyroiditis and Vitamin D Serum Levels

Autoimmune thyroid diseases (AITDs), particularly HT and Graves’ disease (GD), represent the most prevalent organ-specific autoimmune disorders. Most of the evidence linking vitamin D generally supports an association between vitamin D status and the presence of thyroid autoimmunity. Vitamin D has been proposed to play a modulatory role in the hypothalamic–pituitary–thyroid axis, with effects exerted both at the pituitary and thyroid gland levels.

Previous studies have identified the presence of VDR in murine thyrotropic cells, suggesting a potential regulatory role. Moreover, a strong molecular homology between VDR and thyroid hormone receptors has been demonstrated, and VDR expression has been detected in murine thyroid follicular cells. Notably, incubation of these cells with 1,25-dihydroxyvitamin D [1,25(OH)_2_D] resulted in inhibition of both iodine uptake and cell proliferation [44].

Seasonal variations in TSH and vitamin D levels have been observed in euthyroid adults, with TSH peaking during autumn and winter and 25(OH)D levels being highest in spring and summer [45]. An inverse relationship between vitamin D and TSH levels has been consistently reported, alongside a high prevalence of vitamin D deficiency and hypocalcemia in individuals with hypothyroidism [46,47]. Evidence also suggests that thyroid dysfunction linked to iodine excess may manifest only in the presence of vitamin D deficiency, indicating a possible modulatory role of vitamin D in thyroid regulation [48,49]. Patients with HT tend to have lower serum 25(OH)D levels compared to healthy controls, with pronounced deficiency correlating with disease duration and autoantibody titers [50]. Furthermore, serum 25(OH)D is inversely associated with anti-TPO antibody levels, which appear to decrease following vitamin D supplementation in HT patients [51].

From a pathogenic point of view, low serum concentrations impair immune tolerance by driving Th17/Treg imbalance, promoting dendritic cell activation, and enhancing B-cell responses. In experimental models of autoimmune thyroiditis, supplementation with cholecalciferol has been shown to restore Th17/Treg equilibrium and markedly attenuate thyroid tissue damage [52]. Clinical evidence mirrors these findings: meta-analyses of randomized trials demonstrate significant reductions in anti-TPO and anti-Tg antibody levels, alongside improvements in thyroid function, following vitamin D supplementation [53]. Additional data further indicate that vitamin D exerts modulatory effects on cytokine profiles and lymphocyte activity in HT patients [54].

Vitamin D supplementation appears to exert beneficial effects in attenuating disease activity. Its levels have been associated with key proinflammatory cytokines involved in the disease, suggesting a role in its autoimmune mechanisms. Moreover, vitamin D supplementation has been found to exert beneficial immunological effects, particularly by modulating the balance between proinflammatory and regulatory T cells [55,56].

Beyond vitamin D, deficiencies in several micronutrients appear to play a contributory role in the pathogenesis of HT. Selenium (Se), a critical cofactor for antioxidant selenoproteins and deiodinases, has been consistently associated with thyroid autoimmunity. Patients with low Se status often display higher anti-TPO and anti-Tg titers, and selenium supplementation may help reduce these antibody levels [57,58]. Iron deficiency, by impairing TPO and deiodinase activity, has also been linked to a higher risk and greater activity of autoimmune thyroid diseases [59]. Likewise, vitamin B12 deficiency is frequently observed in AITD, most often in the context of concomitant autoimmune or pernicious gastritis [60]. More recently, severely reduced magnesium levels have been associated with autoantibody positivity and hypothyroidism, suggesting another potential micronutrient pathway contributing to HT [61].

Together, these observations highlight the multifactorial role of micronutrient deficiencies in HT, while underscoring vitamin D deficiency as a central and well-documented contributor to disease pathogenesis.

GD, known as diffuse toxic goiter, is the most common cause of hyperthyroidism. Its pathogenesis is complex and not yet fully understood. Two groups have demonstrated that vitamin D inhibits the pathogenesis of GD through the following immunomodulatory mechanisms: (1) vitamin D inhibits the differentiation and maturation of dentritic cells (DCs), decreases the secretion of the proinflammatory cytokines IL-2, IL-23, and IL-12 by DCs, and blocks hyperthyroidism caused by autoimmune responses; (2) vitamin D can directly act on Th1 and Th2 cells, and inhibit the occurrence of autoimmune responses by inhibiting Th1 cells, and by upregulating the activity of Th2 cells and the level of cytokines they secrete; and (3) vitamin D inhibits B cell proliferation, plasma cell differentiation, immunoglobulin secretion, and memory B cell production, and is associated with the onset of GD [62].

Lower serum vitamin D levels have been associated with higher thyrotropin receptor antibody (TRAb) concentrations in patients with GD, suggesting that vitamin D deficiency may contribute to disease onset [63]. Moreover, low 25(OH)D levels have been strongly correlated with an increased risk of GD recurrence following discontinuation of antithyroid drug (ATD) therapy, indicating that vitamin D status may serve as an independent predictor of relapse [64]. However, not all findings are consistent. Some studies reported reduced vitamin D levels in patients with HT but found no significant differences between GD patients and non-AITD controls [65]. Similarly, while Cho et al. observed no clear benefit of daily vitamin D supplementation in preventing GD recurrence within one year after ATD withdrawal, they did note a delayed time to recurrence in patients who achieved sufficient vitamin D levels [66]. Animal studies provide additional insight. In a murine model, BALB/c and C57BL/6 mice were immunized to induce TRAb production while being fed either vitamin D-sufficient or -deficient diets. BALB/c mice, more susceptible to disease induction and less efficient at converting 25(OH)D to 1,25(OH)2D, demonstrated a greater likelihood of developing persistent hyperthyroidism, highlighting the role of genetic background and vitamin D metabolism in modulating autoimmune responses [67].

#### 3.1.4. Bidirectional Relationship Between Autoimmune Thyroiditis and the Main Autoimmune Skin Diseases

Overall, mounting evidence suggests that AIT is closely linked to a spectrum of dermatologic autoimmune disorders, with CSU and vitiligo representing the most consistent associations. In CSU, thyroid autoantibodies are detected far more frequently than in the general population, and their presence often correlates with disease chronicity and severity, suggesting that thyroid autoimmunity may act as an amplifier of skin inflammation [7]. Vitiligo, on the other hand, shares both epidemiological and mechanistic overlaps with AIT: genetic susceptibility loci, altered cytokine profiles, and impaired regulatory T-cell function point to a common immune dysregulation targeting both melanocytes and thyroid tissue [68]. Beyond these two prototypical conditions, other skin disorders such as alopecia areata and psoriasis have also been associated with AIT, albeit less consistently, reinforcing the notion of a systemic autoimmune diathesis that manifests in multiple organs [69]. Taken together, these findings emphasize that skin and thyroid autoimmunity should not be regarded as isolated phenomena, but rather as interconnected facets of the same underlying immune imbalance, warranting integrated diagnostic and therapeutic strategies.

### 3.2. Role of Oxidative Stress in Chronic Spontaneous Urticaria, Hashimoto’s Thyroiditis, and Vitiligo

#### 3.2.1. Correlation Between Chronic Spontaneous Urticaria and Oxidative/Nitrosative Stress

CSU is a complex inflammatory condition, and while its pathogenesis was classically linked to mast cell degranulation and immune dysregulation, recent evidence has highlighted the pivotal roles of OS and NS in both adult and pediatric populations [70].

Several studies have shown elevated oxidative stress markers in CSU patients, such as malondialdehyde (MDA), total oxidant status (TOS), and oxidative stress index (OSI), accompanied by reductions in total antioxidant status (TAS) and enzymatic antioxidants [70,71,72]. Recently, research in pediatric CSU has confirmed these findings, thus establishing a positive correlation between OS markers and urticaria activity scores (UAS7). Moreover, a novel dimension has been added by the demonstration of NS involvement in CSU pathophysiology. A pediatric study reported significantly elevated levels of plasma total nitric oxide metabolites (NOx), nitrite, and nitrate in CSU patients compared to controls [73]. Notably, NOx and nitrite levels correlated positively with disease activity (UAS), whereas nitrate did not. These findings suggest NS as an additional inflammatory pathway contributing to CSU.

Nitric oxide (NO) is a reactive nitrogen species synthesized from L-arginine by nitric oxide synthases (NOS). While constitutive NOS isoforms (eNOS, nNOS) produce low, regulatory NO levels, inducible NOS (iNOS) can generate up to 1000× more NO, particularly during inflammation. iNOS is expressed in macrophages, neutrophils, endothelial and epithelial cells, and is activated during immune responses [74]. High NO levels are proinflammatory, inducing vasodilation, vascular permeability, leukocyte recruitment, and possibly tissue damage-features consistent with CSU pathophysiology. Although histamine is the primary mediator of wheals, NS may contribute independently, especially in patients unresponsive to antihistamines. This is supported by findings of NOS expression in skin inflammatory diseases, including CSU, and iNOS expression in keratinocytes during urticarial episodes [74,75,76].

NOx measurements, including nitrite and nitrate, are promising non-invasive biomarkers of disease activity. NOx was found to have a stronger correlation with UAS than nitrite, thus suggesting that Nox may be a potential objective marker for CSU monitoring, filling a current gap in disease quantification tools [74,75,76].

Beyond ROS and RNS, novel OS-related biomarkers such as advanced oxidation protein products (AOPPs) have been highlighted to be elevated in CSU serum, indicating protein oxidative damage and immune activation. Conversely, advanced glycation end-products (AGEs) were not significantly altered in CSU, limiting their diagnostic utility in this condition [11].

The identification of redox imbalance in CSU opens the door to targeted therapeutic strategies. Agents such as vitamins C and E, N-acetylcysteine, carotenoids, and selenium may offer benefit as adjuvant therapies. In parallel, pharmacologic inhibition of iNOS is being explored in other inflammatory diseases and may hold promise for CSU, particularly in patients with elevated NOx. In animal models of asthma and inflammation, iNOS inhibitors reduce vascular permeability and leukocyte infiltration, effects that may be translatable to CSU if further validated. With hundreds of NOS inhibitor drugs in preclinical or early clinical phases, CSU may become a future candidate for such treatments [74,75,76].

#### 3.2.2. Hashimoto’s Thyroiditis and Oxidative Stress

Due to its role in hormone biosynthesis through oxidative iodination, the thyroid is naturally susceptible to OS. ROS are produced physiologically during thyroid hormone synthesis but are normally buffered by antioxidants such as superoxide dismutase (SOD), glutathione peroxidase (GPx), and catalase (CAT) [77]. In HT patients, this balance is disrupted: evidence consistently shows increased lipid peroxidation, protein oxidation, nitrite and myeloperoxidase levels, along with decreased antioxidant enzyme activity and thiol content [78,79]. Excessive ROS leads to molecular damage affecting nucleic acids, carbohydrates, proteins, and lipids, finally leading to thyrocyte apoptosis and necrosis. In particular, nicotinamide adenine dinucleotide phosphate (NADPH) oxidase has been implicated in excessive ROS generation by overactivated T and B lymphocytes [78]. Moreover, clinical studies have reported elevated serum levels of MDA in HT patients, supporting the presence of systemic OS [80,81,82].

The pathological cascade in HT involves several interconnected mechanisms. Oxidative modifications of thyroglobulin (Tg) and TPO can lead to the formation of neoantigens, which may trigger autoimmune responses [77]. This process is further amplified by ROS, which activate innate immune sensors such as TLRs, promoting Th1 and Th17 immune pathways [78]. Additionally, TSH itself can induce OS, enhancing ROS production and leading to increased protein carbonylation and lipid peroxidation [79]. Antioxidant defenses, including SOD and key micronutrients, like vitamins A, E, and β-carotene, are often depleted, contributing to redox imbalance [79,83]. Moreover, elevated levels of reactive oxygen metabolites and AGEs have been observed, particularly in patients with euthyroid or subclinical hypothyroid forms of the disease, underscoring the clinical relevance of OS in its pathophysiology [83].

Se plays a crucial role in redox regulation through GPx and thioredoxin reductase. Low serum Se levels are commonly observed in HT patients, especially in iodine-rich or Se-deficient regions [84]. Se deficiency led to impaired ROS detoxification and altered Treg/Th1/Th2 balance, enhancing autoimmune activity [85]. Clinical studies have shown that selenium supplementation can improve redox balance and modulate immune responses by reducing IFN-γ and increasing IL-1β. When combined with myo-inositol, selenium also lowers TSH levels and reduces CXCL10, a chemokine linked to lymphocytic infiltration and tissue damage in Hashimoto’s thyroiditis [86].

Se supplementation has been shown to reduce anti-TPO and anti-Tg antibody titers and to improve quality of life, particularly in patients with high antibody levels [12]. In pregnant women, a daily dose of 200 µg of selenomethionine has been associated with a lower risk of postpartum thyroid dysfunction [87]. When combined with levothyroxine, Se may enhance treatment efficacy; however, some trials have reported increased adverse events, likely due to Se overdosing in the absence of baseline status assessment [77]. Despite these promising effects, the variability of results across studies highlights the importance of a personalized approach to selenium therapy, tailored to individual selenium status.

Children and adolescents with HT exhibit increased OS markers, particularly MDA, and reduced antioxidant levels such as Se and GSH, regardless of thyroid function status [77]. Gender differences are notable, with women showing a tenfold higher risk of developing HT [84], potentially due to estrogen-related ROS production and immune activation. Female patients also tend to have lower GPx and SOD activity compared to males [77].

#### 3.2.3. Correlation Between Vitiligo and Oxidative Stress

Among the several etiological factors proposed so far, OS has emerged as a central player in triggering melanocyte destruction and exacerbating vitiligo progression [88]. ROS are natural byproducts of cellular metabolism, including melanogenesis. However, the excessive accumulation of pro-oxidants, including MDA, can overwhelm the antioxidant defense system (CAT, glutathione reductase, GPx, and SOD), finally causing oxidative damage to DNA, protein oxidation, and lipid peroxidation [89]. In vitiligo, multiple studies have demonstrated increased ROS levels in the epidermis, contributing to melanocyte apoptosis and impairing their capacity to counteract oxidative insults [9]. Genetic predisposition has been demonstrated to play a pivotal role in determining an individual’s susceptibility to OS. Several polymorphisms affecting antioxidant enzymes, such as CAT and GPx, have been identified in vitiligo patients, suggesting an inherent vulnerability and predisposition to oxidative damage. Reduced expression or activity of key enzymes results in reduced ability to neutralize ROS, thereby enhancing melanocyte susceptibility to oxidative injury [90]. In addition to direct cellular toxicity, OS in vitiligo seems to trigger and amplify autoimmune responses, which in turn enhance the recruitment of autoreactive T cells and the production of proinflammatory cytokines such as IFN-γ and TNF-α, finally driving the JAK/STAT-mediated melanocyte apoptosis [89]. ROS can be induced by both endogenous and exogenous stressors. Among exogenous triggers, UV radiation and monobenzone, the most used depigmenting agent, are the most representative ones. A central endogenous trigger, on the other hand, is melanin and melanogenesis itself, as it is an energy-intensive mitochondrial process that generates a pro-oxidant state [91]. Mitochondrial dysfunction has been extensively implicated in vitiligo pathogenesis, as these organelles are the primary sites of ROS generation. Mitochondrial dysfunction, including reduced membrane potential and impaired respiratory chain activity, has been observed in vitiligo melanocytes, fostering an environment of heightened oxidative stress. This leads to self-perpetuating cycles of cellular damage, further triggering melanocyte damage and apoptosis [86]. Moreover, OS has been shown to reduce melanocyte adhesiveness at the borders of vitiligo lesions, potentially contributing to the Koebner phenomenon [92]. Simple adhesion molecules, especially integrins and cadherins, mediate melanocyte-keratinocyte interactions without the need for specialized adhesive structures, like desmosomes. In the non-lesional skin of vitiligo patients, E-cadherin expression is reduced, while tenascin, an anti-adhesion molecule, is upregulated [93]. In vitiligo-affected skin, chronic friction can activate epithelial cells, triggering the conversion of mechanical forces into biochemical signals, which in turn leads to intracellular stress and subsequent dysregulation of cadherin expression [94,95]. Additionally, both keratinocytes and melanocytes release chemokines that facilitate T cell recruitment in response to oxidative stress. Overproduction of ROS activates the unfolded protein response (UPR) and causes melanocytes to secrete exosomes that contain melanocyte-specific antigens, micro RNAs (miRNAs), heat shock proteins (HSPs), and damage-associated molecular patterns (DAMPs) [96]. These exosomes transport vitiligo target antigens to nearby DCs and induce their maturation into efficient antigen-presenting cells. UPR activation also induces the production of CXCL16 by stressed keratinocytes, leading to the recruitment of CXCR6+ CD8 T cells; and stressed melanocytes release CXCL12 and CCL5 that are also involved in T cell homing to the skin. CD8+ T cells from vitiligo lesions produce IFN-γ, which in turn activates the JAK-STAT pathway, finally leading to melanocytes’ apoptosis [97]. From its pathogenetic involvement in vitiligo, it derives oxidative stress implications in therapy, with antioxidant-based therapies being explored as potential interventions. Compounds such as vitamin C, vitamin E, polyphenols, and N-acetylcysteine have been investigated for their capacity to scavenge ROS and restore redox homeostasis [98,99].

Among these strategies, NB-UVB phototherapy, widely used in vitiligo, has demonstrated not only immunomodulatory but also antioxidant effects, by reducing ROS production, enhancing activity of protective enzymes, including SOD and CAT, and downregulating proinflammatory cytokine release [100].

**Figure 1:**
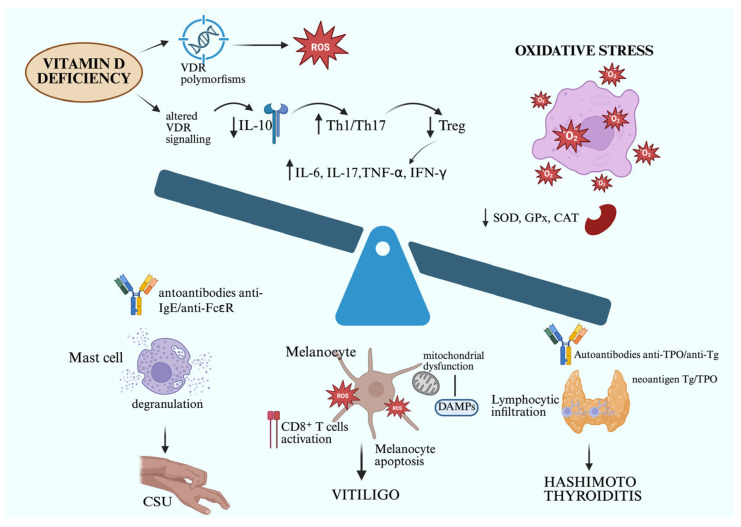
Schematic representation of the shared immunopathogenic pathways linking Hashimoto’s thyroiditis (HT), vitiligo, and chronic spontaneous urticaria (CSU), highlighting the central role of vitamin D deficiency and oxidative stress. Low serum 25(OH)D levels and vitamin D receptor (VDR) polymorphisms impair immune tolerance by reducing regulatory T cell activity and IL-10 secretion, while promoting Th1/Th17 polarization and increased production of proinflammatory cytokines (IL-6, IL-17, TNF-α, IFN-γ). This dysregulated immune background enhances susceptibility to autoimmunity. Concurrently, excessive production of reactive oxygen species (ROS), along with impaired antioxidant defense systems (SOD, GPx, CAT), leads to tissue-specific oxidative damage. In HT, ROS-induced oxidative modifications of thyroglobulin (Tg) and thyroid peroxidase (TPO) generate neoantigens, triggering autoantibody formation (anti-TPO, anti-Tg) and thyrocyte apoptosis. In vitiligo, accumulation of ROS drives mitochondrial dysfunction, melanocyte apoptosis, and release of damage-associated molecular patterns (DAMPs), promoting CD8^+^ T cell–mediated immune responses and depigmentation. In CSU, ROS contribute to mast cell and basophil activation, degranulation, and autoantibody production against IgE or its high-affinity receptor (FcεRI). Together, vitamin D deficiency acts as the unifying upstream factor, while oxidative stress represents the key downstream effector mechanism, converging to sustain chronic immune activation and tissue-specific autoimmunity across these three conditions. Upward arrows indicate an increase in values; downward arrows indicate a decrease in levels. Created in https:// BioRender.com.

Nevertheless, OS plays a pivotal pathogenic role in several other dermatologic conditions, including inflammatory and neoplastic ones. For example, in atopic dermatitis (AD), ROS contribute to skin barrier disruption and exacerbate Th2-driven inflammation, correlating with disease severity. In this context, NB-UVB phototherapy has also been shown to exert antioxidative effects, helping to restore redox equilibrium while controlling inflammation [101]. Similarly, in cutaneous malignancies such as basal cell carcinoma and squamous cell carcinoma, chronic UV-induced OS contributes to DNA damage, mutagenesis, and tumor progression. Interestingly, in this neoplastic setting, photodynamic therapy (PDT) paradoxically utilizes ROS in a controlled and targeted manner to induce tumor cell apoptosis and modulate the tumor immune microenvironment [102]. Thus, while ROS overproduction is a key driver in many cutaneous conditions, controlled modulation of oxidative mechanisms-either by suppression or therapeutic induction-may serve as a rational strategy for disease control. Overall, the persistent imbalance between ROS production and antioxidant defense mechanisms represents a unifying feature across inflammatory and neoplastic skin diseases, underscoring OS as a common molecular pathogenetic mechanism and, thus, promising therapeutic target (Figure 1) [103].

##### Highlights

- Vitiligo, HT, and CSU share common pathogenic mechanisms, including redox imbalance, autoimmunity, and genetic susceptibility.
- OS and NS play a central role across all three conditions, contributing to tissue damage, autoantibody production, and immune system overactivation.
- Vitamin D deficiency is highly prevalent in patients with vitiligo, HT, and CSU, and is associated with increased disease activity and immune dysregulation.
- Vitamin D supplementation improves immune balance, reducing proinflammatory cytokines and enhancing Treg function, supporting its therapeutic role.
- Cross-screening for autoimmune comorbidities among patients with vitiligo, HT, or CSU is recommended to enable earlier diagnosis and patient-tailored management.

### 3.3. Study Limitations

This review has some limitations. Most of the available evidence derives from observational studies, which preclude causal inference. Considerable heterogeneity exists among populations, biomarkers of OS, and definitions of vitamin D deficiency, limiting comparability. As a narrative review, potential selection bias cannot be excluded. Finally, interventional trials on vitamin D or antioxidant supplementation are scarce and report inconsistent results, reducing the strength of therapeutic conclusions.

## 4. Conclusions

The frequent coexistence of CSU, vitiligo, and HT highlights the importance of recognizing polyautoimmunity as a clinical entity with shared immunological, oxidative, and metabolic pathways. Vitamin D deficiency and redox imbalance emerge as central, potentially modifiable contributors to disease pathogenesis. A multidisciplinary approach, including early screening for comorbidities, assessment of vitamin D status, antioxidant support, and targeted therapies, may enhance patient outcomes and guide more personalized treatment strategies.

## Data Availability

Not applicable.
